# Association between inflammatory biomarkers and acute respiratory distress syndrome or acute lung injury risk: A systematic review and meta-analysis

**DOI:** 10.1007/s00508-021-01971-3

**Published:** 2021-12-03

**Authors:** Zhenfeng Liu, Daishun Liu, Zhihua Wang, Yugang Zou, Haixia Wang, Xiao Li, Deliang Zheng, Guoqi Zhou

**Affiliations:** 1 Department of Respiratory Medicine, Zunyi Honghuagang District People’s Hospital, 185 Wanli Road, HongHuagang District, 563000 Guizhou, China; 2 Department of Respiratory Medicine, the Third Affiliated Hospital of Zunyi Medical University, 98 Fenghuang Road, Huichuan District, 563000 Guizhou, China; 3 Department of Respiratory Medicine, Teaching Hospital of Zunyi Medical College, 134 LinJiapo Road, HongHuagang District, 563000 Guizhou, China; 4 Department of Respiratory Medicine, Suzhou Science & Technology Town Hospital, 215153 Jiangsu, China

**Keywords:** Inflammation, Acute lung injury, Acute respiratory distress syndrome, Systematic review, Meta-analysis

## Abstract

**Background:**

The relationship between acute respiratory distress syndrome (ARDS)/acute lung injury (ALI) and levels of certain inflammatory factors remains controversial. The purpose of this meta-analysis was to summarize the available studies evaluating the association between levels of inflammatory factors and ARDS/ALI incidence.

**Methods:**

We searched the PubMed, EmBase, and Cochrane databases for studies published up to July 2017. For each inflammatory factor, a random effects model was employed to pool results from different studies.

**Results:**

We identified 63 studies that included 6243 patients in our meta-analysis. Overall, the results indicated that the levels of angiopoietin (ANG)-2 (standard mean difference, SMD: 1.34; *P* < 0.001), interleukin (IL)-1β (SMD: 0.92; *P* = 0.012), IL‑6 (SMD: 0.66; *P* = 0.005), and tumor necrosis factor (TNF)-α (SMD: 0.98; *P* = 0.001) were significantly higher in patients with ARDS/ALI than in unaffected individuals. No significant differences were observed between patients with ARDS/ALI and unaffected individuals in terms of the levels of IL‑8 (SMD: 0.61; *P* = 0.159), IL-10 (SMD: 1.10; *P* = 0.231), and plasminogen activator inhibitor (PAI)-1 (SMD: 0.70; *P* = 0.060).

**Conclusions:**

ARDS/ALI is associated with a significantly elevated levels of ANG‑2, IL-1β, IL‑6, and TNF‑α, but not with IL‑8, IL-10, and PAI‑1 levels.

**Supplementary Information:**

The online version of this article (10.1007/s00508-021-01971-3) contains supplementary material, which is available to authorized users.

## Background

Acute respiratory distress syndrome (ARDS) and acute lung injury (ALI) are pulmonary diseases characterized by inflammatory pulmonary edema, acute hypoxemia, and accumulation of bilateral pulmonary infiltrates [1]. In the USA the annual number of ARDS cases is more than 140,000 [2] and the reported rates of ARDS and ALI are 59 and 79 cases for every 100,000 individuals, respectively, with reported mortality rates ranging from 22% to 41% [3]. ARDS and ALI are triggered by several factors that are associated with either direct or indirect injury. Direct injuries that trigger ARDS/ALI include serious pulmonary infection, aspiration of foreign bodies, pulmonary contusion, and oxygen poisoning. Indirect injuries associated with ARDS/ALI include severe systemic infection, severe non-pulmonary trauma, acute pancreatitis, major blood transfusion, and disseminated intravascular coagulation [4, 5]. Patients exposed to the abovementioned factors are at high risk for developing ARDS/ALI. Although the mechanisms underlying ARDS/ALI pathogenesis remain unclear, inflammation has been considered as one of the major inducing factors.

The pathology of ARDS is characterized by diffuse pulmonary interstitial and alveolar edema due to capillary endothelial and alveolar epithelial cell injuries that lead to acute respiratory insufficiency or failure [6]. In the early stages of ARDS, edema fluid is concentrated in the alveolar interstitial spaces, and neutrophils adhere to the surface of damaged vascular endothelial cells and migrate to pulmonary interstitial and alveolar cavities. Afterwards, proinflammatory factors, such as inflammatory cytokines and proteases, are released and promote neutrophil-mediated lung injury [7]. In the later stage of ARDS, the injured lung is characterized by severe fibrosis and alveolar destruction and reconstruction [8]. The majority of ARDS/ALI patients show rapid disease progression because of this acute inflammatory process.

Various inflammatory mediators that activate the inflammatory cascade and induce secondary diffuse lung parenchymal injury are the primary causes of ARDS/ALI [9, 10]; however, even low levels of inflammatory factors that do not contribute to the amplification of inflammation are associated with the onset of ARDS/ALI, suggesting their possible role as critical drivers of the disease. Therefore, inflammation-related factors can potentially serve as reliable predictors of ARDS/ALI risk.

A previous study systematically reviewed the relationship between plasma biomarkers and ARDS onset and found that levels of Krebs von den Lungen‑6 (KL-6), lactate dehydrogenase (LDH), the soluble receptor for advanced glycation end products (RAGE), and von Willebrand factor (vWF) were significantly correlated with ARDS incidence [11]; however, the study did not investigate the inflammatory factors present in the pulmonary edema fluid, and conclusions were based on a relatively small number of studies for each inflammatory factor. Therefore, further validation of these putative associations is required. This study provided update results by analyzing recently published literature that investigated the relationships between specific inflammatory biomarkers and the onset of ARDS/ALI. Furthermore, these relationships were analyzed according to different characteristics.

## Methods

We conducted the meta-analysis in accordance with the Preferred Reporting Items for Systematic Reviews and Meta-Analyses (PRISMA) statement [12].

### Search strategy

We systematically searched the PubMed, EmBase, and Cochrane Central Register of Controlled Trials databases for publications up to July 2017 using the keywords “acute respiratory distress syndrome,” “acute lung injury,” “inflammation,” “C-reactive protein,” “interleukin,” “tumour necrosis factor,” “cytokines,” “interferon,” “transforming growth factor,” and “risk factor.” The search strategy used for the PubMed database is described in Supplementary information (searching strategy in PubMed). We restricted our search to reports published in English. We also included relevant articles cited as references of the studies.

### Data selection and extraction

Literature search and selection were independently performed by two researchers, and any inconsistencies were resolved by group discussion. A study was eligible for inclusion if the following criteria were met: (1) the study included patients with ARDS/ALI; (2) participants in the control group were not diagnosed with ARDS/ALI; (3) the primary outcomes of interest included Angiopoietin (ANG)‑2, Interleukin (IL)-1β, IL‑6, IL‑8, IL-10, Plasminogen activator inhibitor (PAI)‑1, and Tumor necrosis factor (TNF)‑α, while the secondary outcomes included albumin, ANG‑1, Clara cell secretory protein (CC16), C-reactive protein (CRP), endotoxin, granulocyte colony-stimulating factor (G‑CSF), intercellular cell adhesion molecule (ICAM), IL‑2, IL‑4, IL-12, KL‑6, Lactate dehydrogenase (LDH), myeloperoxidase (MPO), nuclear factor (NF)-κβ, procalcitonin (PCT), protein C, RAGE, sE-selectin, surfactant protein (SP‑D), transforming growth factor (TGF)-β1, tissue factor (TF), Tumor necrosis factor receptor (TNFR)‑1, TNFR‑2, vascular endothelial growth factor (VEGF), and vWF. Reviews, editorials, non-human studies, letters, and conference papers were excluded because of insufficient data.

The following parameters were extracted from the articles: first author, country, publication year, study design, sample size, and average age, gender, underlying disease of participants, patient disease status, method of ARDS/ALI diagnosis, specimen source, test time, and follow-up. The Newcastle-Ottawa Scale (NOS) was used to evaluate the methodological quality of each study [13]. The NOS is based on the following three subscales: selection of the study group (four categories), comparability of the groups (one category), and outcome assessment (three categories). Data extraction and quality assessment were conducted independently by two authors, and results were examined and adjudicated by an additional author who referred to the original study.

### Statistical analysis

In this meta-analysis, the standard mean difference (SMD) and 95% confidence interval (CI) were determined to evaluate the effect of sample size across studies [14]. We pooled the SMDs for each inflammatory factor using a random effects model [15]. The I^2^ statistic was used to assess heterogeneity of the SMDs across multiple studies [16]. Means and variances were estimated from medians and ranges as previously described [17]. A sensitivity analysis was performed by sequentially removing each individual study from the meta-analysis [16]. Meta-regression was also conducted for ANG‑2, IL-1β, IL6, IL‑8, IL10, PAI‑1, and TNF‑α based on sample size and mean age. Furthermore, stratified analyses were conducted based on sample size, mean age, patient status, and sample origin. Visual inspection of funnel plots from the Egger’s [18] or Begg’s test [19] was conducted to evaluate publication bias. All tests were two-tailed, and *P*-value < 0.05 was considered statistically significant. Data analyses were performed using STATA software (version 10.0; Stata Corporation, College Station, TX, USA).

## Results

### Literature search

**Fig. 1:**
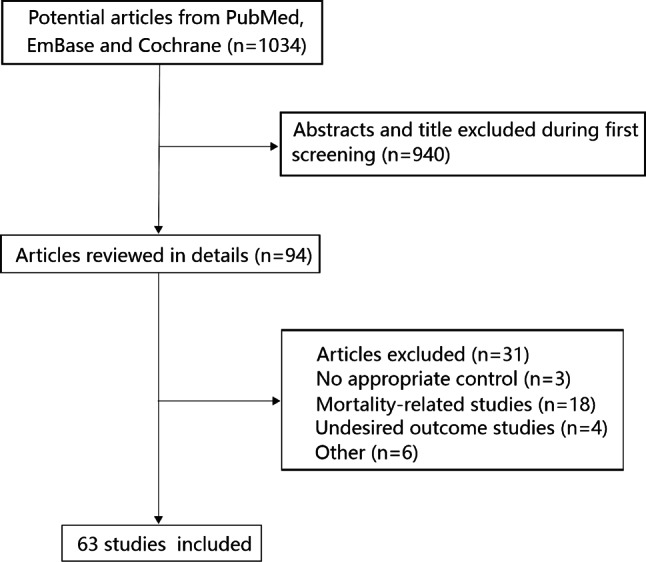
PRISMA flowchart illustrating the process of study selection in our analysis

In this study, a total of 302 articles were retrieved from PubMed, 704 from EmBase, and 28 from the Cochrane Library. After removing duplicates, 851 articles passed the inclusion criteria in the meta-analysis. A total of 757 articles were excluded because they were considered irrelevant after scanning the titles and abstracts. Furthermore, articles that were considered to be unrelated based on full-text assessment (*n* = 5), a duplicate publication (*n* = 1), mortality-related studies (*n* = 18), studies with undesired outcomes (*n* = 4), and studies with control groups without ARDS/ALI risk (*n* = 3) were also excluded. Finally, 63 studies that studied a total of 6243 patients were included in our systematic review (Fig. 1; [20–82]).

### Study characteristics

A total of 59 studies implemented a prospective design, while the remaining 4 studies employed a retrospective design. Most studies included patient groups with an average age ranging from 40 to 60 years. All participants enrolled in the included studies were at risk of ARDS/ALI, and the majority of samples were collected less than 1 day after study recruitment. All included studies employed standardized American-European Consensus Conference (AECC) criteria, Berlin definition of ARDS, lung injury score (LIS), and oxygenation index (PaO2/FiO2) for ARDS/ALI diagnosis. In earlier studies, there were no general criteria for defining ARDS, so the criteria were defined by the authors of each study. The NOS quality analysis for all studies returned scores ranging from 6 to 8, indicating a good overall quality of the included studies (Table 1).

**Table 1:** Characteristics of subjects in eligible studies

Authors	Country	Year	Study design	Sample size	Mean or median age (years)	Gender (m/f)	Underlying disease	Patient status	Judgment method of ARDS/ALI	Specimen source	Test time	Follow-up	NOS score
Hoeboer [12]	Netherlands	2015	Prospective	101	64.0	69/32	Fever	ARDS	Berlin definition/LIS	Blood	7 days	7 days	8
Roubinian [13]	U.S	2015	Prospective	317	58.0	153/164	Pulmonary transfusion reactions hypoxemia	ALI	Defined	Blood	1 day	NA	6
Jones [14]	U.S	2013	Prospective	43	45.6	40/9	Inhalation and burns	ALI	PaO_2_/FiO_2_	BALF	3 days	3 days	7
Agrawal [15]	U.S	2013	Prospective	230	65.0	79/88	Critically ill	ALI	Berlin definition	Blood	1 day	60 days	7
Schultz [16]	Netherlands	2012	Retrospective	20	59.0	13/7	Mechanical ventilation	ALI	LIS	BALF	6 days	6 days	7
Quesnel [17]	France	2012	Retrospective	122	49.0	79/43	Critically ill	ALI/ARDS	AECC	BALF	NA	28 days	6
Osaka [18]	Japan	2011	Prospective	27	50.0	12/15	Pneumonia	ALI	PaO_2_/FiO_2_	Blood	1 day	10 days	8
Guervilly [19]	France	2011	Prospective	74	58.0	53/21	Critically ill	ALI	AECC	Blood/BALF	1 day	28 days	7
Jabaudon [20]	France	2011	Prospective	64	59.0	41/23	Severe Sepsis	ALI	AECC	Blood	1 day	28 days	7
Aman [21]	Netherlands	2010	Prospective	83	60.0	65/18	Mechanical ventilation	ALI/ARDS	AECC	Blood	1 day	NA	7
Kohno [22]	Japan	2011	Prospective	20	71.0	15/5	Thoracic aortic aneurysm repair	ARDS	PaO_2_/FiO_2_	Blood	1–4 days	22 days	7
Determann [23]	Netherlands	2010	Prospective	36	58.0	22/14	Mechanical ventilation	ALI/ARDS	LIS	Blood	2 days	2 days	8
Determann [24]	Netherlands	2010	Prospective	150	61.0	99/51	Mechanical ventilation	ALI	LIS	Blood/BALF	1 day	4 days	8
Fremont [25]	U.S	2010	Retrospective	192	39.0	131/61	Traumatic injuries	ALI	PaO_2_/FiO_2_	Blood	3 days	6–10 days	7
Determann [26]	Netherlands	2009	Retrospective	22	65.0	17/5	Ventilator-associated pneumonia	ALI/ARDS	AECC	BALF	1 day	8 days	7
Chi [27]	China	2009	Prospective	27	NA	NA	Orthotopic liver transplantation	ALI	PaO_2_/FiO_2_	Blood	1 day	7 days	6
Kropski [28]	U.S	2009	Prospective	32	43.0	15/17	Mechanical ventilation	ARDS	AECC	Blood/BALF	1 day	2–14 days	7
Calfee [29]	U.S	2009	Prospective	67	51.0	40/27	Hydrostatic pulmonary edema	ALI	AECC	Blood/BALF	4 h	3 days	8
Van der Heijden [30]	Netherlands	2008	Prospective	112	56.0	NA	Critically ill	ALI/ARDS	AECC/LIS	Blood	1 day	NA	7
Kurzius-Spencer [31]	U.S	2008	Prospective	21	NA	20/1	Smoke inhalation injury	ARDS	AECC/PaO_2_/FiO_2_	BALF	36 h	72 h	8
Nathani [32]	U.K	2008	Prospective	42	62.0	24/18	ARDS risk population	ARDS/ALI	AECC	Blood/BALF	1 day	4 days	7
Ganter [33]	U.S	2008	Prospective	208	41.0	155/53	Traumatic injuries	ALI	AECC	Blood	1 day	28 days	7
Gallagher [34]	U.S	2008	Prospective	63	67.0	35/28	Critically ill	ALI/ARDS	AECC	Blood	1 day	2 months	7
Calfee [35]	U.S	2007	Prospective	1451	52.0	609/839	Trauma	ALI	Defined	Blood	1 day	180 days	7
Ware [36]	U.S	2007	Prospective	878	52.0	514/364	Acute cardiogenic pulmonary edema	ALI/ARDS	Defined	Blood	1 day	3 days	7
Perkins [37]	UK	2007	Prospective	54	NA	NA	ARDS risk population	ALI/ARDS	AECC	BALF	1 day	4 days	6
El Solh [38]	U.S	2006	Prospective	51	36.6	22/29	Aspiration pneumonitis	ALI	AECC	Blood/BALF	1 day	28 days	8
Parsons [39]	U.S	2005	Prospective	49	NA	NA	Critically ill	ALI	AECC	Blood	1 day	180 days	8
Bouros [40]	Greece	2004	Prospective	59	51.7	43/16	Critically ill	ALI	AECC	Blood/BALF	1 day	NA	7
Nakae [41]	Japan	2003	Prospective	21	62.0	15/6	Sepsis	ARDS	Defined	Blood	NA	NA	7
Sato [42]	UK	2004	Prospective	37	39.5	32/5	Mechanical ventilation	ARDS	AECC	Blood	1 day	6.5 days	8
Nys [43]	Belgium	2003	Prospective	67	54.0	43/24	Pneumonia	ARDS	PaO_2_/FiO_2_	BALF	1–2 days	NA	7
Gessner [44]	Germany	2003	Prospective	35	60.0	16/19	Acute respiratory failure	ARDS	AECC	BALF	1 day	6 months	7
Ishizaka [45]	Japan	2004	Prospective	35	68.0	27/8	Cardiogenic pulmonary edema	ALI	AECC	BALF	1 day	NA	6
Prabhakaran [46]	U.S	2003	Prospective	51	50.0	29/22	Hydrostatic edema	ARDS	AECC	Blood/BALF	1 day	NA	7
Grissom [47]	U.S	2003	Prospective	39	51.0	16/17	ARDS risk population	ARDS	AECC	Blood/BALF	96 h	42 days	8
Agouridakis [48]	Greece	2002	Prospective	65	44.0	40/25	Mechanical ventilation	ARDS	AECC	Blood/BALF	1 day	15 days	8
Agouridakis [49]	Greece	2002	Prospective	34	49.0	23/11	Mechanical ventilation	ARDS	AECC	Blood/BALF	1 day	6 months	8
Thickett [50]	UK	2002	Prospective	68	65.0	45/23	ARDS risk population	ARDS	AECC	BALF	1 day	4 days	6
Hamacher [51]	France	2002	Prospective	36	43.0	28/8	ARDS risk population	ARDS	AECC	BALF	1 day	21 days	7
Takala [52]	Finland	2002	Prospective	52	54.0	29/19	Critically ill	ARDS	AECC	Blood	1 day	7 days	8
Park [53]	Switzerland	2001	Prospective	69	43.8	41/28	ARDS risk population	ARDS	AECC	BALF	1 day	21 days	7
Hirani [54]	UK	2001	Prospective	56	48.0	NA	Major trauma	ARDS	AECC	BALF	1 day	36 months	8
Geerts [55]	Belgium	2001	Prospective	26	52.0	19/7	ARDS risk population	ARDS	AECC	BALF	1 day	NA	7
Siemiatkowski [56]	Poland	2000	Prospective	36	44.3	27/9	Major trauma	ARDS	LIS	Blood	1 day	10 days	7
Armstrong [57]	UK	2000	Prospective	67	62.0	44/23	Critically ill	ARDS/ALI	AECC	BALF	48 h	NA	7
Bauer [58]	Spain	2000	Prospective	66	57.2	NA	Pneumonia	ARDS	AECC	Blood	1 day	NA	8
Gando [59]	Japan	1999	Prospective	58	58.0	37/21	Critically ill	ARDS	Defined	Blood	1 day	4 days	7
Donnelly [60]	Scotland	1999	Prospective	61	NA	NA	Trauma	ARDS	Defined	BALF	NA	NA	6
Parsons [61]	U.S	1997	Prospective	77	37.5	53/24	ARDS risk population	ARDS	Defined	Blood	1 day	2 days	7
Schutte [62]	Germany	1996	Prospective	56	54.5	45/11	Pneumonia	ARDS	AECC	Blood/BALF	2 days	10 days	7
Chollet-Martin [63]	France	1996	Prospective	14	61.0	NA	Pneumonia	ARDS	LIS	Blood/BALF	3 days	7 days	8
Ricou [64]	U.S	1996	Prospective	33	48.0	24/9	Critically ill	ARDS	LIS	Blood/BALF	3 days	2 weeks	8
Schwartz [65]	U.S	1996	Prospective	12	45.0	7/5	Mechanical ventilation	ARDS	LIS	BALF	1 day	NA	7
Jorens [66]	UK	1995	Prospective	35	56.6	31/4	Cardiopulmonary bypass	ARDS	Defined	BALF	1 day	3 days	7
Fuchs-buder [67]	Switzerland	1996	Prospective	21	NA	NA	Critically ill	ARDS	LIS	BALF	2 days	10 days	8
Leff [68]	U.S	1993	Prospective	26	NA	NA	Sepsis	ARDS	Defined	Blood	1 day	2 days	7
Sakamaki [69]	Japan	1995	Prospective	48	49.0	29/19	Sepsis	ARDS	Defined	Blood	1 day	15 days	7
Donnelly [70]	U.S	1994	Prospective	82	49.5	NA	ARDS risk population	ARDS	LIS	Blood	1–3 days	NA	8
Moalli [71]	U.S	1989	Prospective	47	66.0	NA	ARDS risk population	ARDS	Defined	Blood	1 day	22 days	7
Rubin [72]	U.S	1990	Prospective	45	NA	NA	Sepsis	ARDS	LIS	Blood	1 day	3 days	8
Roten [73]	Switzerland	1990	Prospective	50	49.0	31/19	Critically ill	ARDS	Defined	Blood	1 day	5 days	7
Parsons [74]	U.S	1992	Prospective	103	46.0	77/26	ARDS risk population	ARDS	Defined	Blood	1 day	2 days	7

*AECC* American-European Consensus Conference, *BALF* Bronchoalveolar Lavage Fluid, *ALI* Acute lung injury, *ARDS* Acute respiratory distress syndrome, *NA* not available

### Inflammatory biomarkers and ARDS/ALI

**Fig. 2:**
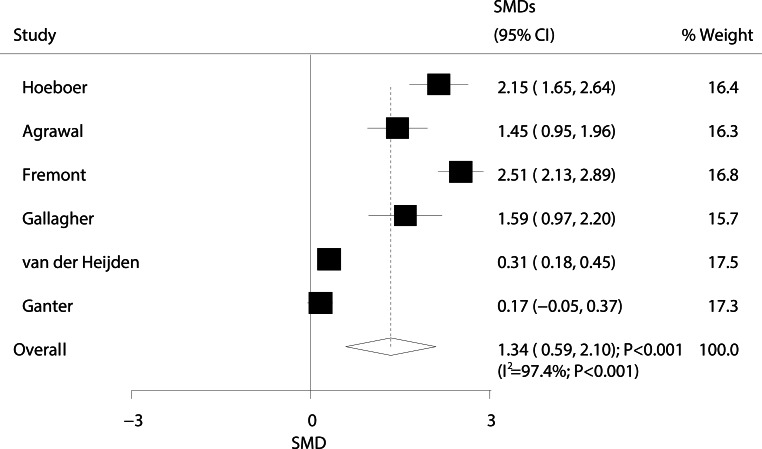
Forest plot comparing ANG‑2 levels between ARDS/ALI patients and unaffected individuals

The relationship between ARDS/ALI and angiopoietin (ANG)-2 levels is presented in Fig. 2. The overall standard mean difference (SMD) from six studies showed that ARDS/ALI patients had higher ANG‑2 levels than those of unaffected individuals (SMD: 1.34; 95% CI: 0.59–2.10; *P* < 0.001); however, significant heterogeneity was detected (I^2^ = 97.4%; *P* < 0.001). Sensitivity analysis showed that the conclusion did not change after sequential removal of each study (Supplementary information Table S1).

**Fig. 3:**
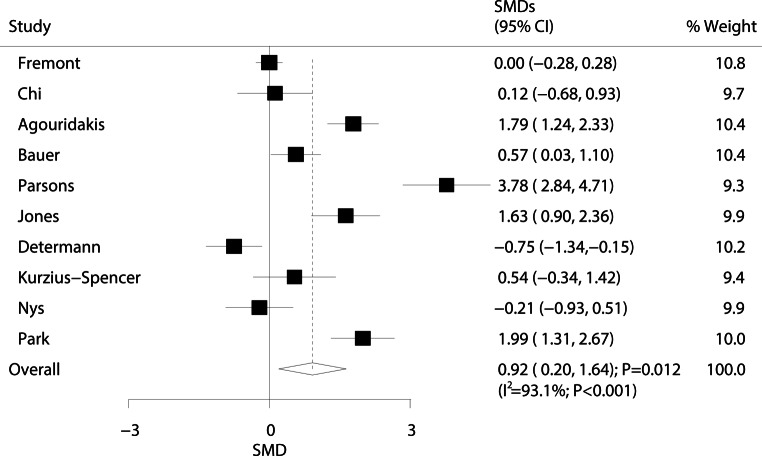
Forest plot comparing IL-1β levels between ARDS/ALI patients and unaffected individuals

The relationship between ARDS/ALI and interleukin (IL)-1β levels is presented in Fig. 3. The pooled SMD from 10 studies indicated that ARDS/ALI patients exhibited significantly higher IL-1β levels than those of the individuals without ARDS/ALI (SMD: 0.92; 95% CI: 0.20–1.64; *P* = 0.012). Although substantial heterogeneity was observed across all studies (I^2^ = 93.1%; *P* < 0.001), the conclusion did not change after sequential exclusion of each study (Supplementary information Table S2).

**Fig. 4:**
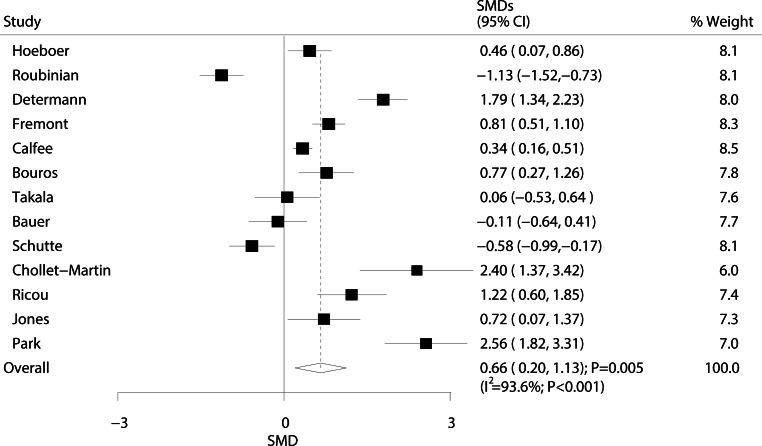
Forest plot comparing IL‑6 levels between ARDS/ALI patients and unaffected individuals

The relationship between ARDS/ALI and IL‑6 levels is shown in Fig. 4. Overall results showed that ARDS/ALI patients had higher IL‑6 levels than those of individuals in the population without ARDS/ALI (SMD: 0.66; 95% CI: 0.20 to 1.13; *P* = 0.005). Heterogeneity was observed at the same degree as the effect across the studies (I^2^ = 93.6%; *P* < 0.001). Sensitivity analysis showed that the conclusion was not affected by the exclusion of any specific study from the pooled analysis (Supplement information Table S3).

**Fig. 5:**
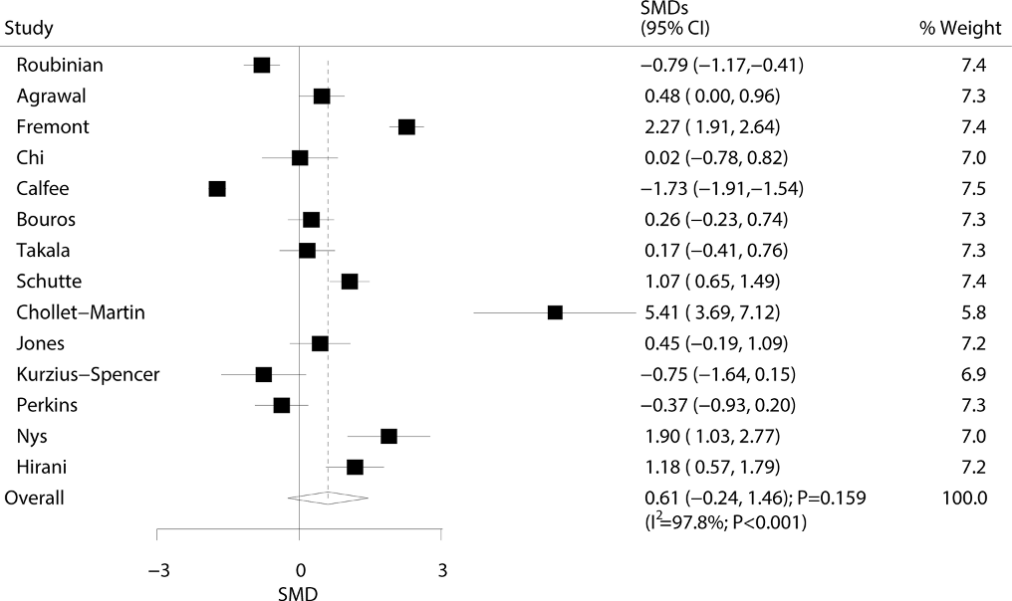
Forest plot comparing IL‑8 levels between ARDS/ALI patients and unaffected individuals

The relationship between ARDS/ALI and IL‑8 levels was analyzed in 14 studies, and results are shown in Fig. 5. No significant differences in IL‑8 levels were observed between ARDS/ALI patients and individuals of the population without ARDS/ALI (SMD: 0.61; 95% CI: −0.24–1.46; *P* = 0.159). Furthermore, substantial heterogeneity was detected (I^2^ = 97.8%; *P* < 0.001). Based on sensitivity analysis, we excluded the study conducted by Calfee et al. [57], which specifically included a large sample size of trauma patients. We concluded that ARDS/ALI were associated with higher IL‑8 levels (SMD: 0.76; 95% CI: 0.11–1.40; *P* = 0.021) (Supplement information Table S4).

**Fig. 6:**
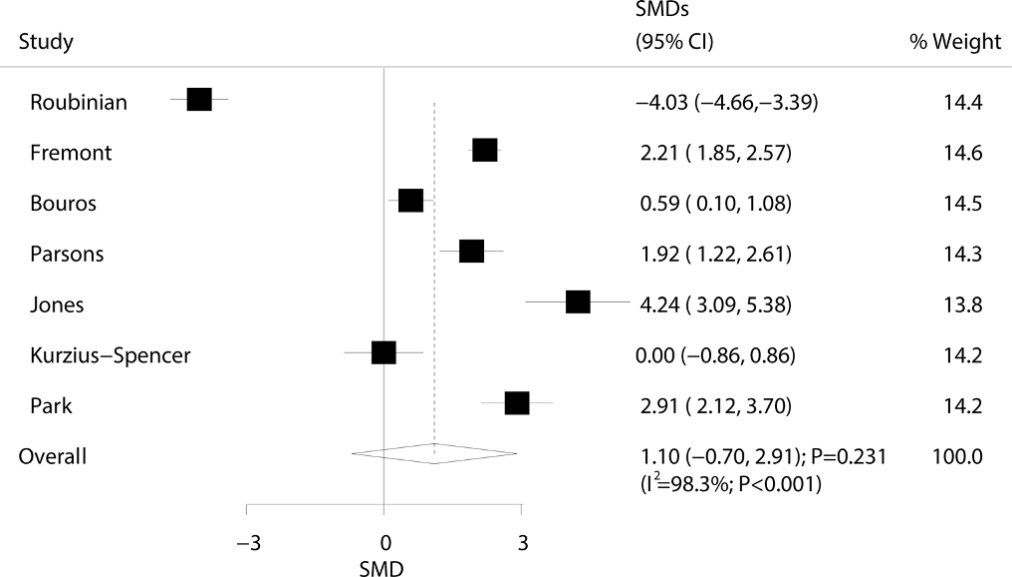
Forest plot comparing IL-10 levels between ARDS/ALI patients and unaffected individuals

The relationship between ARDS/ALI and IL-10 levels was investigated in seven studies, and results are presented in Fig. 6. We detected no significant differences in IL-10 levels between ARDS/ALI and non-ARDS/ALI patients (SMD: 1.10; 95% CI: −0.70–2.91; *P* = 0.231). Substantial heterogeneity was observed (I^2^ = 98.3%; *P* < 0.001). Sensitivity analysis indicated that ARDS/ALI patients had higher IL-10 levels than those of non-ARDS/ALI patients when the study conducted by Roubinian et al. was excluded (Supplementary information Table S5) [82].

**Fig. 7:**
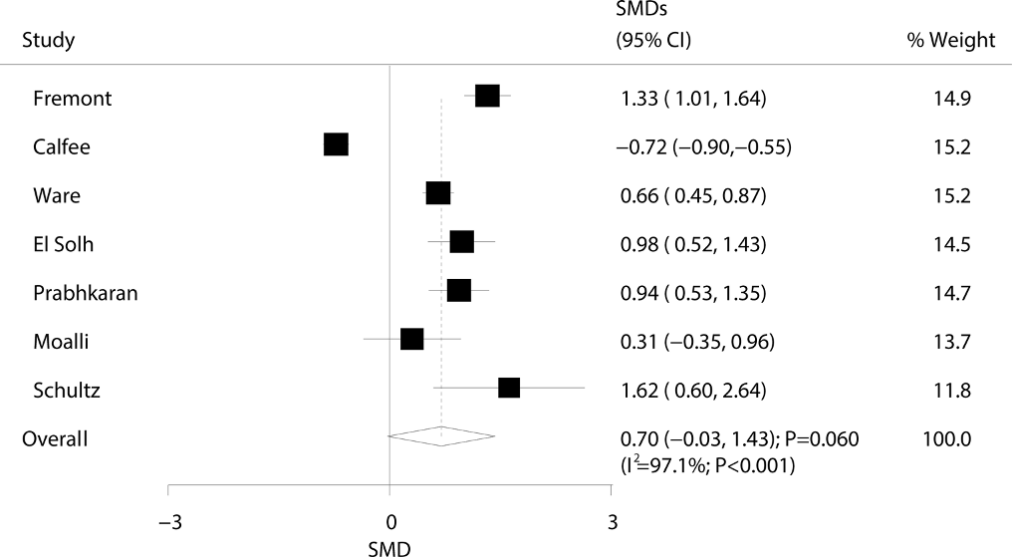
Forest plot comparing PAI‑1 levels between ARDS/ALI patients and unaffected individuals

The relationship between ARDS/ALI and plasminogen activator inhibitor‑1 (PAI-1) levels was investigated in seven studies, and results are presented in Fig. 7. We detected no significant differences in PAI‑1 levels between ARDS/ALI patients and non-ARDS/ALI individuals (SMD: 0.70; 95% CI: −0.03–1.43; *P* = 0.060). Substantial heterogeneity was observed (I^2^ = 97.1%; *P* < 0.001). Sensitivity analysis showed that this result changed after excluding the study conducted by Calfee et al. (Supplementary information Table S6) [57].

**Fig. 8:**
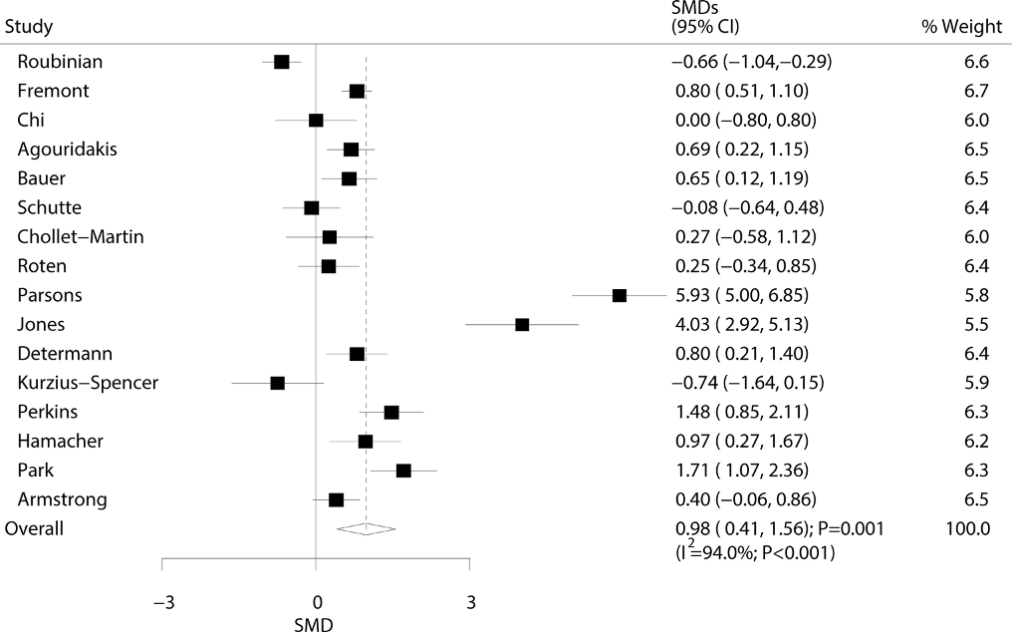
Forest plot comparing TNF‑α levels between ARDS/ALI patients and unaffected individuals

The relationship between ARDS/ALI and tumour necrosis factor (TNF)-α levels was investigated in 16 studies, and results are shown in Fig. 8. Pooled results showed that ARDS/ALI patients had significantly higher TNF‑α levels than those of individuals without ARDS/ALI (SMD: 0.98; 95% CI: 0.41–1.56; *P* = 0.001). Significant heterogeneity was detected across all included studies (I^2^ = 94.0%; *P* < 0.001). These results did not change after sequential exclusion of any specific study (Supplementary information Table S7).

The correlations between ARDS/ALI and other inflammatory factors based on sample origin are summarized in Table 2. Overall, ARDS/ALI patients showed higher levels of albumin (SMD: 2.15; *P* = 0.010), ANG‑1 (SMD: 4.60; *P* < 0.001), KL‑6 (SMD: 2.23; *P* = 0.044), myeloperoxidase (MPO) (SMD: 1.75; *P* < 0.001), transforming growth factor (TGF)-β1 (SMD: 0.83; *P* = 0.013), transfer factor (TF) (SMD: 5.57; *P* < 0.001), and TNF receptor‑1 (SMD: 5.40; *P* < 0.001). Moreover, ARDS/ALI patients had lower levels of IL-12 (SMD: −1.47; *P* < 0.001), surfactant protein D (SP-D) (SMD: −1.17; *P* = 0.012), and vascular endothelial growth factor (VEGF) (SMD: −4.52; *P* < 0.001) in the bronchial alveolar lavage fluid (BALF). In addition, ARDS/ALI patients had higher levels of KL‑6 (SMD: 3.36; *P* < 0.001), MPO (SMD: 2.58; *P* < 0.001), procalcitonin (PCT) (SMD: 0.41; *P* = 0.038), receptor for advanced glycation end products (RAGE) (SMD: 1.64; *P* = 0.031), sE-selectin (SMD: 0.55; *P* = 0.011), TF (SMD: 3.55; *P* < 0.001), and TNF receptor‑2 (SMD: 3.82; *P* < 0.001) than unaffected individuals. ARDS/ALI was associated with lower IL-12 (SMD: −0.80; *P* < 0.001) levels in the blood. No other significant differences were observed between ARDS/ALI and non-ARDS/ALI patients.

**Table 2:** Summary of results of the association of other inflammatory factors with ARDS/ALI based on specimen source

Factors	No. of studies	Groups	SMD	95% CI	*P* value	Heterogeneity (%)	*P* for heterogeneity
Albumin	1	Blood	−0.82	−1.79 to 0.14	0.095	**–**	**–**
	**2**	**BALF**	**2.15**	**0.51 to 3.79**	**0.010**	**82.2**	**0.018**
ANG‑1	1	Blood	0.80	0.28 to 2.30	0.676	**–**	**–**
	**1**	**BALF**	**4.60**	**3.09 to 6.12**	**<** **0.001**	–	–
CC16	3	Blood	−0.31	−2.7 to 2.08	0.799	96.8	< 0.001
	4	BALF	−0.44	−3.06 to 2.18	0.742	96.1	< 0.001
CRP	3	Blood	1.64	−0.31 to 3.59	0.100	92.2	< 0.001
Endotoxin	2	BALF	0.30	−0.15 to 0.75	0.191	0.0	0.887
G‑CSF	2	Blood	−0.49	−1.47 to 0.49	0.326	93.9	< 0.001
ICAM	4	Blood	−0.14	−2.47 to 2.20	0.909	99.4	< 0.001
	2	BALF	0.79	−1.61 to 3.18	0.520	96.3	< 0.001
IL‑2	2	Blood	0.01	−0.26 to 0.28	0.934	0.0	0.817
	1	BALF	−0.36	−1.16 to 0.44	0.380	**–**	**–**
IL‑4	2	Blood	0.69	−0.16 to 1.54	0.111	81.1	0.022
	1	BALF	0.30	−0.38 to 0.99	0.387	**–**	**–**
IL-12	**1**	**Blood**	**−0.8**	**−1.09 to −0.50**	**<** **0.001**	–	–
	**1**	**BALF**	**−1.47**	**−2.18 to −0.75**	**<** **0.001**	–	–
KL‑6	**3**	**Blood**	**3.36**	**2.50 to 4.21**	**<** **0.001**	**49.5**	**0.138**
	**3**	**BALF**	**2.23**	**0.06 to 4.41**	**0.044**	**93.0**	**<** **0.001**
LDH	2	Blood	1.82	−0.23 to 3.87	0.082	85.9	< 0.001
MPO	**1**	**Blood**	**2.58**	**2.20 to 2.97**	**<** **0.001**	–	–
	**1**	**BALF**	**1.75**	**0.90 to 2.60**	**<** **0.001**	–	–
NF-κβ	2	BALF	0.86	−0.45 to 2.17	0.198	67.1	0.081
PCT	**2**	**Blood**	**0.41**	**0.02 to 0.80**	**0.038**	**23.7**	**0.252**
Protein C	2	Blood	−2.00	−7.15 to 3.16	0.447	99.9	< 0.001
RAGE	**4**	**Blood**	**1.64**	**0.15 to 3.14**	**0.031**	**96.3**	**<** **0.001**
	1	BALF	0.16	−0.68 to 1.00	0.704	–	–
sE-selectin	**3**	**Blood**	**0.55**	**0.13 to 0.97**	**0.011**	**15.2**	**0.307**
SP‑D	4	Blood	−0.05	−1.65 to 1.55	0.950	98.5	< 0.001
	**1**	**BALF**	**−1.17**	**−2.08 to −0.25**	**0.012**	–	–
TGF-β1	**4**	**BALF**	**0.83**	**0.17 to 1.49**	**0.013**	**81.2**	**<** **0.001**
TF	**1**	**Blood**	**3.55**	**2.71 to 4.39**	**<** **0.001**	–	–
	**1**	**BALF**	**5.57**	**3.55 to 7.60**	**<** **0.001**	–	–
TNFR‑1	2	Blood	1.61	−4.42 to 7.64	0.601	98.9	< 0.001
	**1**	**BALF**	**5.40**	**4.22 to 6.58**	**<** **0.001**	–	–
TNFR‑2	**1**	**Blood**	**3.82**	**2.80 to 4.83**	**<** **0.001**	–	–
	2	BALF	3.22	−2.62 to 9.05	0.280	98.4	< 0.001
VEGF	1	Blood	0.49	−0.23 to 1.04	0.063	–	–
	**1**	**BALF**	**−4.52**	**−5.43 to −3.61**	**<** **0.001**	**–**	**–**
vWF	6	Blood	0.81	−0.94 to 2.54	0.365	99.2	< 0.001

*ANG‑1* Angiopoietin‑1, *CC16* Clara cell secretory protein, *CRP* C-reactive protein, *G‑CSF* granulocyte colony-stimulating factor, *ICAM* intercellular cell adhesion molecule, *IL‑2* interleukin‑2, *LDH* Lactate dehydrogenase, *MPO* myeloperoxidase, *NF-κβ* nuclear factor-κβ, *PCT* procalcitonin, *RAGE* receptor for advanced glycation end products, *SP‑D* surfactant protein, *TGF-β1* transforming growth factor-β1, *TF* tissue factor, *TNFR‑1* Tumor necrosis factor receptor‑1, *TNFR‑2* Tumor necrosis factor receptor‑2, *VEGF* vascular endothelial growth factor, *vWF* von Willebrand factor

### Meta-regression and subgroup analyses

A relatively large heterogeneity was observed among the studies included in our meta-analysis. We therefore performed a meta-regression analysis for ANG‑2, IL-1β, IL‑6, IL‑8, IL-10, PAI‑1, and TNF‑α; results are presented in Supplementary information Figures S1–S14. Overall, sample size was determined to influence the association between PAI‑1 levels and ARDS/ALI (*P* = 0.025); no other significant associations were observed. Subgroup analyses were also conducted based on sample size, mean age, patient status, and sample source (Table 3). First, ARDS/ALI did not show a significant influence on ANG‑2 levels when the mean age was < 60.0 years, and patients with ALI. Second, ARDS/ALI was not associated with IL-1β levels if the study sample size was ≥ 100, patients with ALI, or samples were collected from the BALF. Third, no significant associations were detected between ARDS/ALI and IL‑6 levels when the study sample size was ≥ 100, the mean age was < 60.0 years, patients with ALI, and samples were collected from the blood. Fourth, ARDS/ALI were associated with higher IL‑8 levels if the study sample size was < 100, patients had ARDS, or samples were collected from BALF. Fifth, ARDS/ALI were significantly associated with higher IL-10 levels when the study sample size was < 100, patients with ARDS, and samples were collected from the BALF. Sixth, ARDS/ALI patients showed significantly higher PAI‑1 levels when the study sample size was < 100, patients with ARDS or ARDS/ALI, and samples were collected from the BALF. Finally, ARDS/ALI were not associated with TNF‑α levels if the study sample size was ≥ 100, patients with ALI or ARDS/ALI.

**Table 3:** Subgroup analysis of inflammatory related factors and incidence of ARDS/ALI

Inflammatory factors	Groups	No. of studies	SMD	95%CI	*P* value	Heterogeneity (%)	*P* for heterogeneity
ANG‑2	Sample size						
	≥ 100	5	1.30	0.47 to 2.13	0.002	97.8	< 0.001
	< 100	1	1.59	0.97 to 2.20	< 0.001	–	–
	Mean age (years)						
	≥ 60.0	3	1.74	1.30 to 2.18	< 0.001	51.1	0.129
	< 60.0	3	0.98	−0.02 to 1.98	0.055	98.4	< 0.001
	Patients’ status						
	ARDS	1	2.15	1.65 to 2.64	< 0.001	–	–
	ALI	3	1.38	−0.19 to 2.93	0.085	98.3	< 0.001
	ARDS/ALI	2	0.91	−0.34 to 2.16	0.152	93.7	< 0.001
	Specimen source						
	Blood	6	1.34	0.59 to 2.10	< 0.001	97.4	< 0.001
	BALF	0	–	–	–	–	–
IL-1β	Sample size						
	≥ 100	2	−0.33	−1.05 to 0.40	0.379	79.7	0.026
	< 100	8	1.26	0.48 to 2.04	0.002	89.9	< 0.001
	Mean age (years)						
	≥ 60.0	1	−0.75	−1.34 to −0.15	0.014	–	–
	< 60.0	7	1.33	0.43 to 2.23	0.004	94.4	< 0.001
	Patients’ status						
	ARDS	6	1.39	0.41 to 2.37	0.005	91.8	< 0.001
	ALI	4	0.22	−0.59 to 1.04	0.590	88.0	< 0.001
	Specimen source						
	Blood	5	1.39	0.15 to 2.62	0.028	95.3	< 0.001
	BALF	6	0.73	−0.19 to 1.66	0.121	90.2	< 0.001
IL‑6	Sample size						
	≥ 100	5	0.45	−0.26 to 1.16	0.215	96.1	< 0.001
	< 100	8	0.83	0.11 to 1.55	0.024	91.6	< 0.001
	Mean age (years)						
	≥ 60.0	3	1.49	0.37 to 2.61	0.009	92.1	< 0.001
	< 60.0	10	0.43	−0.06 to 0.92	0.088	93.1	< 0.001
	Patients’ status						
	ARDS	7	0.80	0.03 to 1.57	0.043	92.6	< 0.001
	ALI	6	0.54	−0.12 to 1.20	0.108	95.3	< 0.001
	Specimen source						
	Blood	11	0.37	−0.04 to 0.77	0.074	88.8	< 0.001
	BALF	7	1.33	0.24 to 2.42	0.016	93.2	< 0.001
IL‑8	Sample size						
	≥ 100	4	0.06	−1.84 to 1.95	0.953	99.2	< 0.001
	< 100	10	0.74	0.15 to 1.33	0.014	87.7	< 0.001
	Mean age (years)						
	≥ 60.0	2	2.87	−1.95 to 7.70	0.243	96.6	< 0.001
	< 60.0	9	0.52	−0.61 to 1.65	0.364	98.5	< 0.001
	Patients’ status						
	ARDS	6	1.28	0.34 to 2.21	0.007	90.4	< 0.001
	ALI	7	0.13	−1.13 to 1.40	0.834	98.6	< 0.001
	ARDS/ALI	1	−0.37	−0.93 to 0.20	0.201	–	–
	Specimen source						
	Blood	9	0.67	−0.50 to 1.85	0.262	98.3	< 0.001
	BALF	8	0.78	0.08 to 1.48	0.029	86.1	< 0.001
IL-10	Sample size						
	≥ 100	2	−0.90	−7.01 to 5.21	0.772	99.6	< 0.001
	< 100	5	1.89	0.59 to 3.19	0.004	93.4	< 0.001
	Mean age (years)						
	≥ 60.0	0	–	–	–	–	–
	< 60.0	6	1.29	−0.75 to 3.32	0.216	98.6	< 0.001
	Patients’ status						
	ARDS	3	1.62	0.05 to 3.19	0.043	91.7	< 0.001
	ALI	4	0.73	−2.15 to 3.61	0.618	99.0	< 0.001
	Specimen source						
	Blood	4	0.18	−2.64 to 3.00	0.899	99.0	< 0.001
	BALF	4	1.90	0.12 to 3.68	0.037	94.3	< 0.001
PAI‑1	Sample size						
	≥ 100	3	0.42	−0.78 to 1.61	0.497	98.8	< 0.001
	< 100	4	0.89	0.52 to 1.27	< 0.001	42.1	0.159
	Mean age (years)						
	≥ 60.0	1	0.31	−0.35 to 0.96	0.363	–	–
	< 60.0	6	0.77	−0.04 to 1.57	0.063	97.6	< 0.001
	Patients’ status						
	ARDS	2	0.68	0.06 to 1.29	0.030	61.1	0.109
	ALI	4	0.77	−0.54 to 2.08	0.250	98.1	< 0.001
	ARDS/ALI	1	0.66	0.45 to 0.87	< 0.001	–	–
	Specimen source						
	Blood	6	0.44	−0.34 to 1.22	0.267	97.2	< 0.001
	BALF	3	1.57	0.77 to 2.36	< 0.001	69.6	0.037
TNF‑α	Sample size						
	≥ 100	4	1.66	−0.10 to 3.43	0.065	98.3	< 0.001
	< 100	12	0.76	0.27 to 1.24	0.002	85.5	< 0.001
	Mean age (years)						
	≥ 60.0	3	0.51	0.17 to 0.84	0.003	0.0	0.482
	< 60.0	10	1.36	0.52 to 2.20	0.002	96.0	< 0.001
	Patients’ status						
	ARDS	9	1.05	0.11 to 1.99	0.028	94.7	< 0.001
	ALI	5	0.90	−0.14 to 1.95	0.090	95.1	< 0.001
	ARDS/ALI	2	0.92	−0.14 to 1.97	0.089	86.5	0.007
	Specimen source						
	Blood	9	1.00	0.12 to 1.89	0.026	95.7	< 0.001
	BALF	10	0.91	0.26 to 1.57	0.006	87.6	< 0.001

### Publication bias

Funnel plots of inflammatory factors and ARDS/ALI incidence are presented in Supplementary information Figures S15–S21. No significant publication biases were detected between ARDS/ALI and IL-1β (*P*-value for Egger’s test (P_Egger_): 0.148; *P*-value for Begg’s test (P_Begg_): 0.283), IL‑6 (P_Egger_: 0.330; P_Begg_: 0.161), IL-10 (P_Egger_: 0.874; P_Egger_: 1.000), PAI‑1 (P_Egger_: 0.184; P_Begg_: 0.548), and TNF‑α (P_Egger_: 0.111; P_Begg_: 0.224). Although results of the Begg’s tests showed no evidence of publication bias for ANG‑2 (*P* = 0.707) and IL‑8 (*P* = 0.827), results of Egger’s test showed potential publication bias (*P*-value for ANG-2: 0.048; *P*-value for IL-8: 0.013). Conclusions did not change after correction using the trim and fill method [83].

## Discussion

In our study, ARDS/ALI were found to be associated with higher levels of ANG‑2, IL-1β, IL‑6, and TNF‑α, whereas no significant associations were detected between ARDS/ALI and IL‑8, IL-10, and PAI‑1 levels. Furthermore, serum levels of KL‑6, MPO, RAGE, sE-selectin, TF, and TNF receptor‑2 were significantly higher in ARDS/ALI patients than in unaffected individuals; however, ARDS/ALI patients had lower IL-12 levels. The BALF concentrations of albumin, ANG‑1, KL‑6, MPO, TGF-β1, TF, and TNF receptor‑1, were significantly higher in ARDS/ALI patients than in individuals without ARDS/ALI. In addition, ARDS/ALI were associated with lower levels of IL-12 and VEGF; however, heterogeneity among studies was substantial, and the amount of data available was insufficient. Therefore, more research is needed to verify the results of our meta-analysis.

Current treatment for ARDS/ALI consists of respiratory support and immunological treatment. Evidence suggests that the dynamic balance between proinflammatory and anti-inflammatory factors plays a key role in the pathogenesis and prognosis of ARDS/ALI [84]; however, cytokine interactions are highly complex and difficult to study. When proinflammatory and anti-inflammatory factors are unbalanced, excess inflammatory cytokines are released, which in turn damage the lung tissues or even whole body tissues. Therefore, studies that investigate inflammatory factors present during the onset of ARDS/ALI can help elucidate the mechanisms underlying ARDS/ALI pathogenesis and serve as the basis for the development of new treatment approaches for ARDS/ALI.

ANG‑2 is a proinflammatory cytokine and a member of the vascular growth factor family. ANG‑2 mainly promotes cell apoptosis and disrupts vascularization and can also act in conjunction with VEGF to promote neovascularization [85, 86]. The findings of the present study indicated that serum levels of ANG‑2 were significantly higher in ARDS/ALI patients than in unaffected individuals. Similar to our current findings, serum levels of ANG‑2 have been associated with other diseases, such as sepsis and pulmonary hypertension [87]. In particular, ANG‑2 serum levels were associated with the onset of septic shock, and ANG‑2 blood concentrations have been observed to increase during endothelial cell inflammation. Furthermore, elevated ANG‑2 levels in the blood are known to promote vascular permeability and leakage; however, only a small number of studies have explored the relationship between ANG‑2 and ARDS/ALI incidence, and the overall results might have been altered by more recent findings from subsequent studies.

Our current results demonstrated that ARDS/ALI patients had significantly higher serum levels of IL-1β than individuals without ARDS/ALI. IL-1β is synthesized and released by mononuclear macrophages. It is recognized as the primary proinflammatory cytokine that triggers inflammation and is known to exert multiple biological functions, such as promoting the activity of natural killer cells, increasing chemotaxis of macrophages and neutrophils, and regulating the immune response as an endogenous heat source [88, 89]. During infection or sepsis, IL-1β can destroy the blood-brain barrier and increase the risk of patient mortality. IL-1β has an inherent antagonist in the human body, IL-1ra, which can inhibit IL-1β activity by competitively binding to its receptor. One study included in this analysis showed that IL-1ra is significantly upregulated in ARDS patients [41]. Anakinra is an IL-1β antagonist approved by the U.S. Food and Drug Administration (FDA) for the treatment of rheumatoid arthritis and other autoimmune diseases to reduce clinical symptoms and suppress joint destruction [90]. Determining whether this antagonistic effect can also be observed in ARDS patients is an interesting topic for future research.

IL‑6 is an acute inflammatory mediator that is released by various cell types. IL‑6 is not expressed under normal physiological conditions but is secreted upon stimulation by inflammatory factors [91]. Our study showed that IL‑6 concentrations in the BALF were significantly higher in ARDS/ALI patients than in unaffected individuals. Consistent with these results, IL‑6 is recognized as a reliable and objective indicator of local lung tissue damage.

IL‑8 is a member of the C-X-C subfamily of chemokines and is produced by various cell types [92]. IL‑8 plays a significant role in neutrophil chemotaxis and also inhibits neutrophil apoptosis. In response to local lung tissue injury, IL‑8 specifically binds to its receptor, which in turn induces neutrophil aggregation and triggers the release of proteolytic enzymes that mediate inflammation and severe tissue damage. Our findings suggested that IL‑8 concentrations are associated with ARDS/ALI incidence.

IL-10 is an anti-inflammatory cytokine that inhibits the secretion of TNF‑α, IL‑1, and IL‑6 [93]. IL-10 can also suppress NF-κβ activity and regulate the Janus kinase-signal transducer and activator of transcription (JAK-STAT) pathway. Our analysis suggested that IL-10 levels are correlated with ARDS/ALI incidence; however, the elevated IL-10 levels could have been caused by inflammation in the lungs.

PAI‑1 is mainly secreted by vascular endothelial cells, and its production is a risk factor for thrombosis and atherosclerosis [94]. During ARDS pathogenesis, the coagulation fibrinolysis system is impaired, leading to disseminated intravascular coagulation. Increased PAI‑1 levels may lead to local fibrin deposition in lungs. Our results support the idea that PAI‑1 plays an important role in the development of ARDS. In addition, PAI‑1 has been demonstrated to promote local formation of diseased connective tissue and has been used as an indicator of prognosis of ARDS patients.

The results of our study suggested a strong correlation between TNF‑α concentrations in the BALF and ARDS incidence. TNF‑α is considered as one of the most important proinflammatory factors in ARDS/ALI [95]. TNF‑α is a multifunctional proinflammatory factor that stimulates the secretion of endothelin and nitric oxide by endothelial cells, promotes the expression of adhesion molecules by endothelial cells and leukocytes, and contributes to the progression of severe microcirculatory disorder. Therefore, TNF‑α inhibition can potentially serve as an important approach for ARDS prevention and treatment.

The general objective of this study was to identify inflammatory factors that can serve as drug targets to reduce the incidence of ARDS/ALI. Multiple lines of evidence have suggested that proinflammatory cytokines participate in or trigger the inflammatory response in the lungs; however, currently available clinical data are insufficient to verify the correlations between the levels of proinflammatory factors and ARDS/ALI incidence.

Our current meta-analysis has several limitations First, results were based on other studies but not at the individual level. Second, the included studies showed significant heterogeneity, making it difficult to eliminate alternative explanations for the results, such as differences in the definition of ARDS/ALI, severity of disease, underlying diseases, sample collection times, and treatment strategies. Third, unpublished articles and articles written in other languages were not searched, which could have skewed the obtained results.

## Conclusion

The results of our study indicated that ARDS/ALI are associated with elevated levels of ANG‑2, IL-1β, IL‑6, and TNF‑α, but do not significantly affect IL‑8, IL-10, and PAI‑1 levels. Furthermore, ARDS/ALI incidence was also determined to be significantly associated with several other inflammatory factors; however, further studies using large sample sizes are required to verify our conclusions. Future studies should also measure the levels of inflammatory factors over time. Log transformation of the measures of inflammatory factors is recommended to obtain a normally distributed data, especially in studies with small sample sizes.

## Supplementary Information

Supplementary information Figures S1–S21. Meta-regression and publication bias of inflammatory biomarkers with acute respiratory distress syndrome or acute lung injury

## Abbreviations

ALI: Acute lung injury
ANG: Angiopoietin
ARDS: Acute respiratory distress syndrome
IL: Interleukin
KL: Krebs von den Lungen
LDH: Lactate dehydrogenase
PAI: Plasminogen activator inhibitor
RAGE: Receptor for advanced glycation end products
SMD: Standard mean difference
TNF: Tumor necrosis factor
vWF: von Willebrand factor
