# Supplementary material for: Association between inflammatory biomarkers and acute respiratory distress syndrome or acute lung injury risk: A systematic review and meta-analysis
Source: Wien Klin Wochenschr. 2021 Dec 3;134(1-2):24–38. doi: 10.1007/s00508-021-01971-3 (PMC8813738; doi:10.1007/s00508-021-01971-3)
Supplement: Supplementary file 1 — Supplementary information Figures S1–S21. Meta-regression and publication bias of inflammatory biomarkers with acute respiratory distress syndrome or acute lung injury [file 508_2021_1971_MOESM1_ESM.docx]

Association between inflammatory biomarkers and acute respiratory distress syndrome or acute lung injury risk: A systematic review and meta-analysis

Table S1. Sensitive analysis of ANG-2

| Excluding study | SMD | 95%CI | P value | Heterogeneity (%) | P value for heterogeneity |
| --- | --- | --- | --- | --- | --- |
| Hoeboer | 1.18 | 0.41 to 1.96 | 0.003 | 97.3 | <0.001 |
| Agrawal | 1.32 | 0.48 to 2.16 | 0.002 | 97.8 | <0.001 |
| Fremont | 1.08 | 0.47 to 1.70 | 0.001 | 95.3 | <0.001 |
| Gallagher | 1.30 | 0.47 to 2.13 | 0.002 | 97.8 | <0.001 |
| van der Heijden | 1.57 | 0.48 to 2.65 | 0.005 | 97.3 | <0.001 |
| Ganter | 1.59 | 0.52 to 2.67 | 0.004 | 97.6 | <0.001 |

Table S2. Sensitive analysis of IL-1β

| Excluding study | SMD | 95%CI | P value | Heterogeneity (%) | P value for heterogeneity |
| --- | --- | --- | --- | --- | --- |
| Fremont | 1.04 | 0.21 to 1.86 | 0.014 | 92.4 | <0.001 |
| Chi | 1.01 | 0.23 to 1.79 | 0.011 | 93.8 | <0.001 |
| Agouridakis | 0.82 | 0.07 to 1.57 | 0.032 | 92.6 | <0.001 |
| Bauer | 0.97 | 0.15 to 1.79 | 0.021 | 93.8 | <0.001 |
| Parsons | 0.63 | 0.01 to 1.25 | 0.048 | 90.3 | <0.001 |
| Jones | 0.84 | 0.08 to 1.61 | 0.030 | 93.4 | <0.001 |
| Determann | 1.11 | 0.37 to 1.85 | 0.003 | 92.7 | <0.001 |
| Kurzius-Spencer | 0.96 | 0.18 to 1.74 | 0.015 | 93.8 | <0.001 |
| Nys | 1.05 | 0.27 to 1.82 | 0.008 | 93.6 | <0.001 |
| Park | 0.80 | 0.06 to 1.54 | 0.034 | 92.8 | <0.001 |

Table S3. Sensitive analysis of IL-6

| Excluding study | SMD | 95%CI | P value | Heterogeneity (%) | P value for heterogeneity |
| --- | --- | --- | --- | --- | --- |
| Hoeboer | 0.69 | 0.18 to 1.19 | 0.008 | 94.1 | <0.001 |
| Roubinian | 0.81 | 0.39 to 1.22 | <0.001 | 91.0 | <0.001 |
| Determann | 0.56 | 0.11 to 1.01 | 0.015 | 92.5 | <0.001 |
| Fremont | 0.66 | 0.14 to 1.17 | 0.013 | 93.8 | <0.001 |
| Calfee | 0.71 | 0.13 to 1.29 | 0.016 | 94.1 | <0.001 |
| Bouros | 0.66 | 0.16 to 1.15 | 0.009 | 94.0 | <0.001 |
| Takala | 0.71 | 0.22 to 1.21 | 0.004 | 94.0 | <0.001 |
| Bauer | 0.73 | 0.24 to 1.22 | 0.004 | 94.0 | <0.001 |
| Schutte | 0.77 | 0.30 to 1.24 | 0.001 | 93.2 | <0.001 |
| Chollet-Martin | 0.55 | 0.09 to 1.01 | 0.020 | 93.6 | <0.001 |
| Ricou | 0.62 | 0.14 to 1.10 | 0.012 | 93.9 | <0.001 |
| Jones | 0.66 | 0.17 to 1.15 | 0.008 | 94.1 | <0.001 |
| Park | 0.51 | 0.07 to 0.96 | 0.024 | 92.8 | <0.001 |

Table S4. Sensitive analysis of IL-8

| Excluding study | SMD | 95%CI | P value | Heterogeneity (%) | P value for heterogeneity |
| --- | --- | --- | --- | --- | --- |
| Roubinian | 0.73 | -0.21 to 1.67 | 0.128 | 97.9 | <0.001 |
| Agrawal | 0.63 | -0.29 to 1.54 | 0.180 | 97.9 | <0.001 |
| Fremont | 0.45 | -0.31 to 1.22 | 0.242 | 96.7 | <0.001 |
| Chi | 0.66 | -0.24 to 1.55 | 0.150 | 97.9 | <0.001 |
| **Calfee** | **0.76** | **0.11 to 1.40** | **0.021** | **94.1** | **<0.001** |
| Bouros | 0.64 | -0.27 to 1.56 | 0.169 | 97.9 | <0.001 |
| Takala | 0.65 | -0.26 to 1.56 | 0.161 | 97.9 | <0.001 |
| Schutte | 0.58 | -0.32 to 1.48 | 0.209 | 97.8 | <0.001 |
| Chollet-Martin | 0.32 | -0.53 to 1.16 | 0.463 | 97.8 | <0.001 |
| Jones | 0.63 | -0.27 to 1.53 | 0.173 | 97.9 | <0.001 |
| Kurzius-Spencer | 0.71 | -0.18 to 1.61 | 0.117 | 97.9 | <0.001 |
| Perkins | 0.69 | -0.22 to 1.60 | 0.137 | 97.9 | <0.001 |
| Nys | 0.51 | -0.36 to 1.39 | 0.250 | 97.8 | <0.001 |
| Hirani | 0.57 | -0.32 to 1.46 | 0.211 | 97.8 | <0.001 |

Table S5. Sensitive analysis of IL-10

| Excluding study | SMD | 95%CI | P value | Heterogeneity (%) | P value for heterogeneity |
| --- | --- | --- | --- | --- | --- |
| Roubinian | 1.93 | 0.96 to 2.90 | <0.001 | 92.9 | <0.001 |
| Fremont | 0.92 | -1.30 to 3.14 | 0.417 | 98.3 | <0.001 |
| Bouros | 1.19 | -1.08 to 3.47 | 0.303 | 98.6 | <0.001 |
| Parsons | 0.97 | -1.13 to 3.06 | 0.365 | 98.5 | <0.001 |
| Jones | 0.60 | -1.32 to 2.52 | 0.539 | 98.4 | <0.001 |
| Kurzius-Spencer | 1.29 | -0.75 to 3.32 | 0.216 | 98.6 | <0.001 |
| Park | 0.80 | -1.20 to 2.81 | 0.433 | 98.5 | <0.001 |

Table S6. Sensitive analysis of PAI-1

| Excluding study | SMD | 95%CI | P value | Heterogeneity (%) | P value for heterogeneity |
| --- | --- | --- | --- | --- | --- |
| Fremont | 0.59 | -0.17 to 1.35 | 0.131 | 96.7 | <0.001 |
| Calfee | 0.93 | 0.61 to 1.25 | <0.001 | 70.8 | 0.004 |
| Ware | 0.72 | -0.23 to 1.66 | 0.137 | 97.2 | <0.001 |
| El Solh | 0.66 | -0.16 to 1.47 | 0.114 | 97.4 | <0.001 |
| Prabhkaran | 0.66 | -0.16 to 1.48 | 0.113 | 97.4 | <0.001 |
| Moalli | 0.77 | -0.04 to 1.57 | 0.063 | 97.6 | <0.001 |
| Schultz | 0.58 | -0.20 to 1.35 | 0.144 | 97.5 | <0.001 |

Table S7. Sensitive analysis of TNF-α

| Excluding study | SMD | 95%CI | P value | Heterogeneity (%) | P value for heterogeneity |
| --- | --- | --- | --- | --- | --- |
| Roubinian | 1.10 | 0.53 to 1.67 | <0.001 | 92.8 | <0.001 |
| Fremont | 1.01 | 0.34 to 1.67 | 0.003 | 94.3 | <0.001 |
| Chi | 1.05 | 0.44 to 1.65 | 0.001 | 94.3 | <0.001 |
| Agouridakis | 1.01 | 0.38 to 1.64 | 0.002 | 94.4 | <0.001 |
| Bauer | 1.01 | 0.39 to 1.63 | 0.001 | 94.4 | <0.001 |
| Schutte | 1.06 | 0.45 to 1.67 | 0.001 | 94.2 | <0.001 |
| Chollet-Martin | 1.03 | 0.43 to 1.64 | 0.001 | 94.4 | <0.001 |
| Roten | 1.04 | 0.42 to 1.65 | 0.001 | 94.3 | <0.001 |
| Parsons | 0.66 | 0.23 to 1.08 | 0.002 | 88.4 | <0.001 |
| Jones | 0.80 | 0.25 to 1.36 | 0.004 | 93.4 | <0.001 |
| Determann | 1.00 | 0.38 to 1.61 | 0.001 | 94.4 | <0.001 |
| Kurzius-Spencer | 1.09 | 0.50 to 1.68 | <0.001 | 94.1 | <0.001 |
| Perkins | 0.95 | 0.35 to 1.56 | 0.002 | 94.2 | <0.001 |
| Hamacher | 0.99 | 0.38 to 1.60 | 0.001 | 94.3 | <0.001 |
| Park | 0.94 | 0.34 to 1.54 | 0.002 | 94.1 | <0.001 |
| Armstrong | 1.03 | 0.40 to 1.66 | 0.001 | 94.3 | <0.001 |


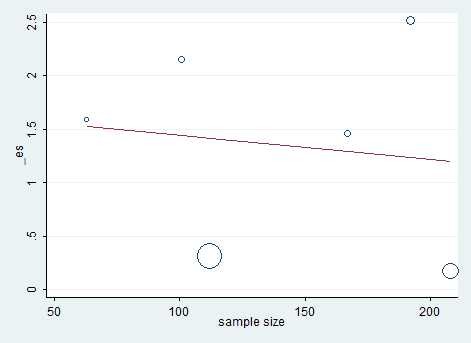


Figure S1. Meta-regression of ANG-2 based on sample size (P=0.800)


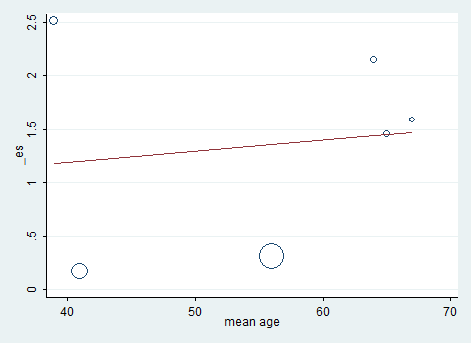


Figure S2. Meta-regression of ANG-2 based on mean age (P=0.801)


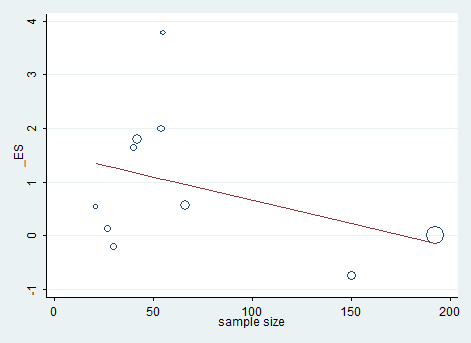


Figure S3. Meta-regression of IL-1β based on sample size (P=0.284)


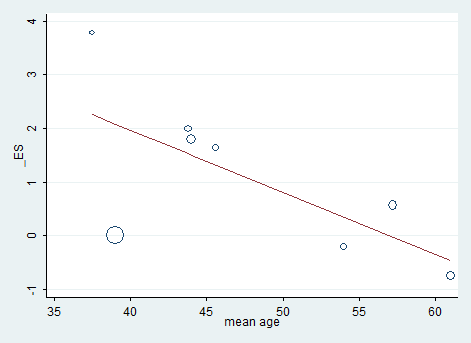


Figure S4. Meta-regression of IL-1β based on mean age (P=0.063)


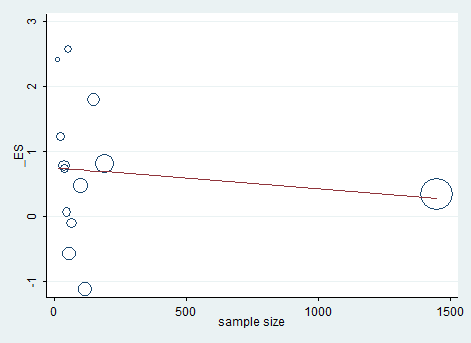


Figure S5. Meta-regression of IL-6 based on sample size (P=0.698)


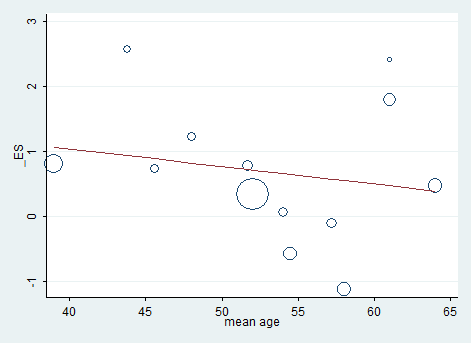


Figure S6. Meta-regression of IL-6 based on mean age (P=0.539)


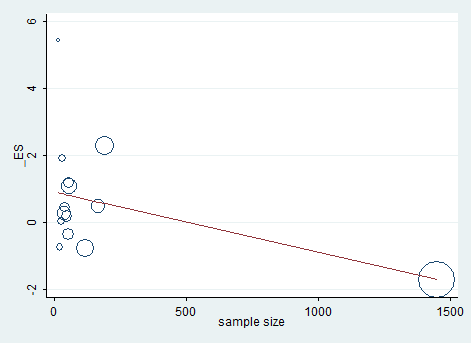


Figure S7. Meta-regression of IL-8 based on sample size (P=0.131)


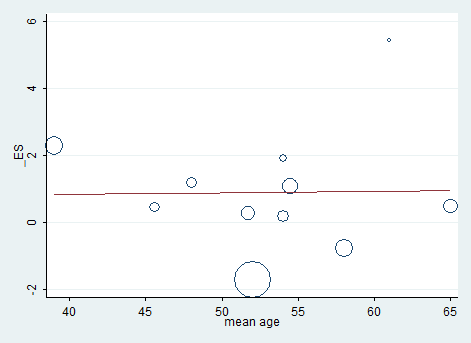


Figure S8. Meta-regression of IL-8 based on mean age (P=0.954)


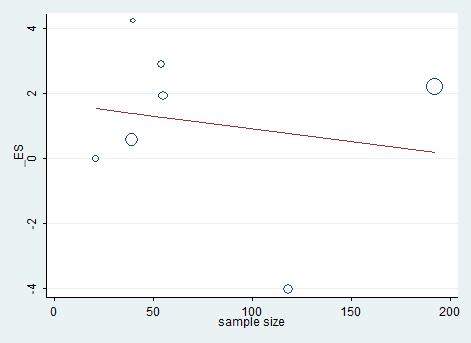


Figure S9. Meta-regression of IL-10 based on sample size (P=0.700)


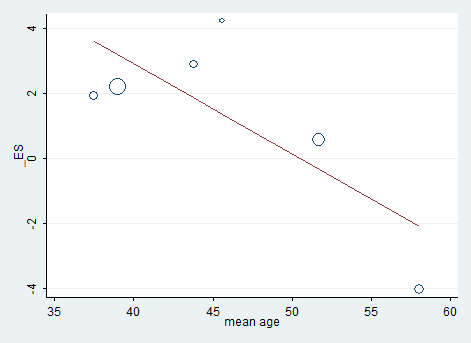


Figure S10. Meta-regression of IL-10 based on mean age (P=0.081)


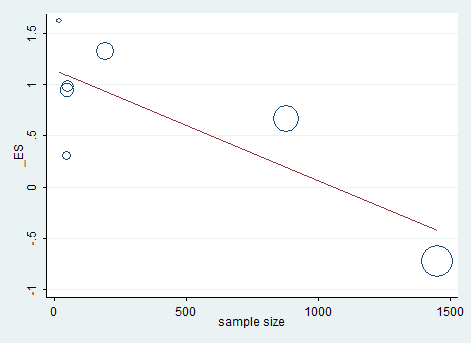


Figure S11. Meta-regression of PAI-1 based on sample size (P=0.025)


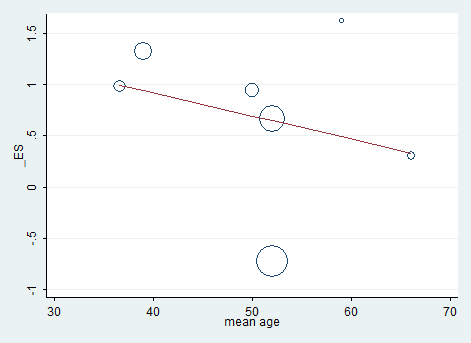


Figure S12. Meta-regression of AI-1 based on mean age (P=0.517)


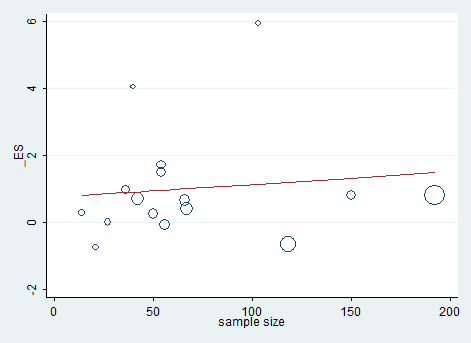


Figure S13. Meta-regression of TNF-α based on sample size (P=0.681)


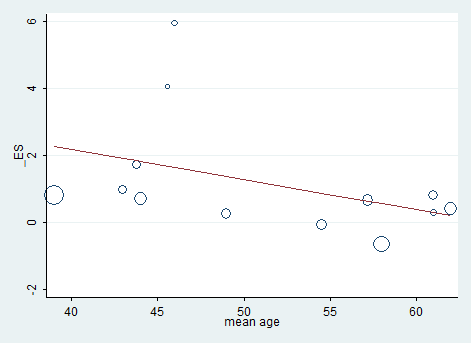


Figure S14. Meta-regression of TNF-α based on mean age (P=0.159)


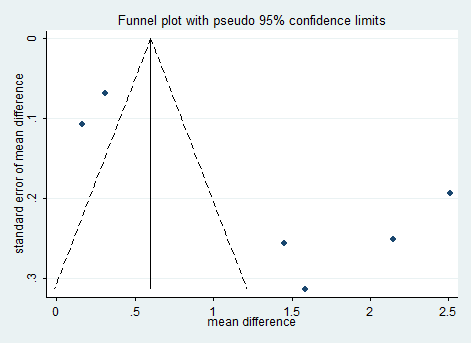


Figure S15. Funnel plot of ANG-2 level and risk of ARDS/ALI (P value for Egger: 0.048; P value for Begg: 0.707)


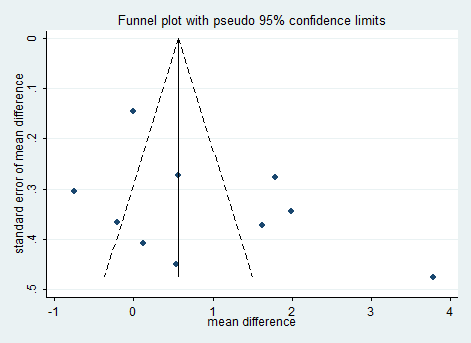


Figure S16. Funnel plot of IL-1β level and risk of ARDS/ALI (P value for Egger: 0.148; P value for Begg: 0.283)


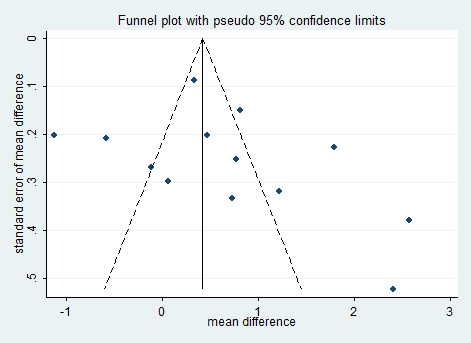


Figure S17. Funnel plot of IL-6 and risk of ARDS/ALI (P value for Egger: 0.330; P value for Begg: 0.161)


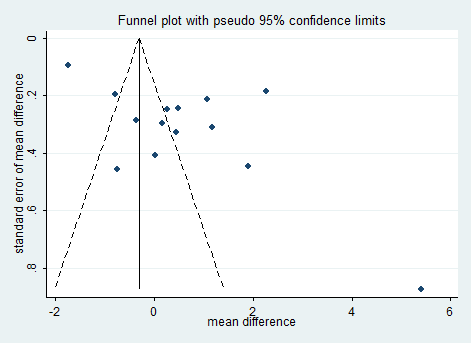


Figure S18. Funnel plot of IL-8 and risk of ARDS/ALI (P value for Egger: 0.013; P value for Begg: 0.827)


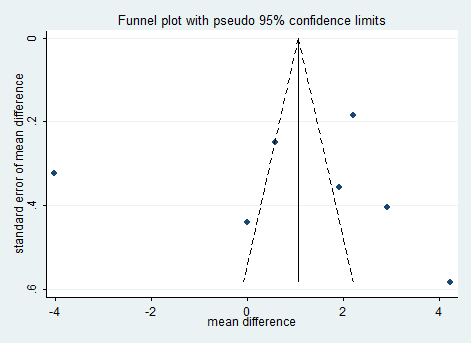


Figure S19. Funnel plot of IL-10 and risk of ARDS/ALI (P value for Egger: 0.874; P value for Begg: 1.000)


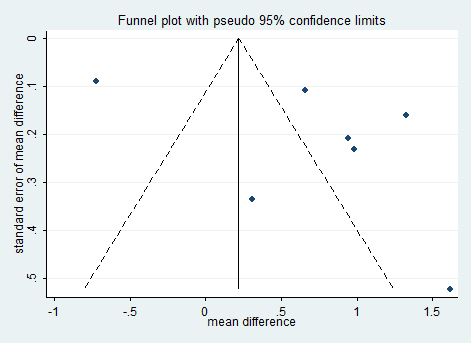


Figure S20. Funnel plot of PAI-1 and risk of ARDS/ALI (P value for Egger: 0.184; P value for Begg: 0.548)


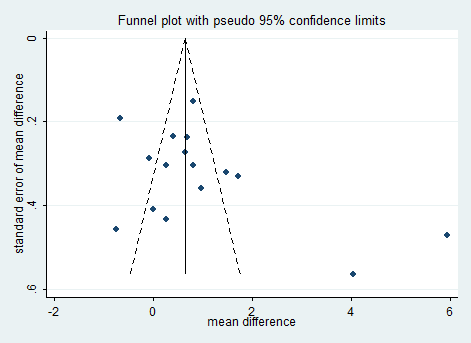


Figure S21. Funnel plot of TNF-α and risk of ARDS/ALI (P value for Egger: 0.111; P value for Begg: 0.224)

**Searching strategy in PubMed**

#1. exp Respiratory Distress Syndrome, Adult/

#2. Adult Respiratory Distress Syndrome/

#3. Acute Lung Injury/

#4. Acute Respiratory Distress Syndrome/

#5. ARDS.mp. or ALI.mp.

#6. #1 or #2 or #3 or #4 or #5

#7. exp systemic inflammatory response syndrome/

#8. systemic inflammatory response syndrome.tw./ or

#9. Inflammation/

#10. C-reactive protein/

#11. Interleukin/

#12. tumour necrosis factor/

#13. Cytokines/

#14. Interferon/

#15. Transforming growth factor/

#16. Risk factor/

#17. #7 or #8 or #9 or #10 or #11 or #12 or #13 or #14 or #15 or #16

#18. #6 and #17
